# Global Statement on Air Pollution and Health: Opportunities for Africa

**DOI:** 10.5334/aogh.2667

**Published:** 2019-12-16

**Authors:** Caradee Y. Wright, Angela Mathee, Stuart Piketh, Kristy Langerman, Tafadzwa Makonese, Siyavuya Bulani, Himla Soodyall

**Affiliations:** 1 Environment and Health Research Unit, South African Medical Research Council, Pretoria, ZA; 2 Department of Geography, Geoinformatics and Meteorology, University of Pretoria, Pretoria, ZA; 3 Environment and Health Research Unit, South African Medical Research Unit, Johannesburg, ZA; 4 Faculty of Health Sciences, University of Johannesburg, ZA; 5 University of the Witwatersrand, Johannesburg, ZA; 6 School of Geo- and Spatial Sciences, North-West University, Potchefstroom, ZA; 7 Department of Geography, Environmental Management and Energy Studies, University of Johannesburg, Johannesburg, ZA; 8 Sustainable Energy Technology and Research Centre, University of Johannesburg, Johannesburg, ZA; 9 Academy of Science of South Africa, Pretoria, ZA

## Abstract

The editorial speaks to the Global Statement on Air Pollution and Health and How it may assist African countries to eliminate air pollution-related health impacts.

## Introduction

The human health and economic costs of air pollution in Africa are high and rising. Between 1990 and 2013, deaths from ambient particulate matter on the African continent rose by more than one third, and by 2013 was costing the African economy approximately USD 215 billion annually [1]. Similarly, premature deaths associated with domestic fuel combustion rose by 18% between 1990 and 2013 and cost the African economy approximately USD 232 billion in 2013 [1].

**Figure 1:**
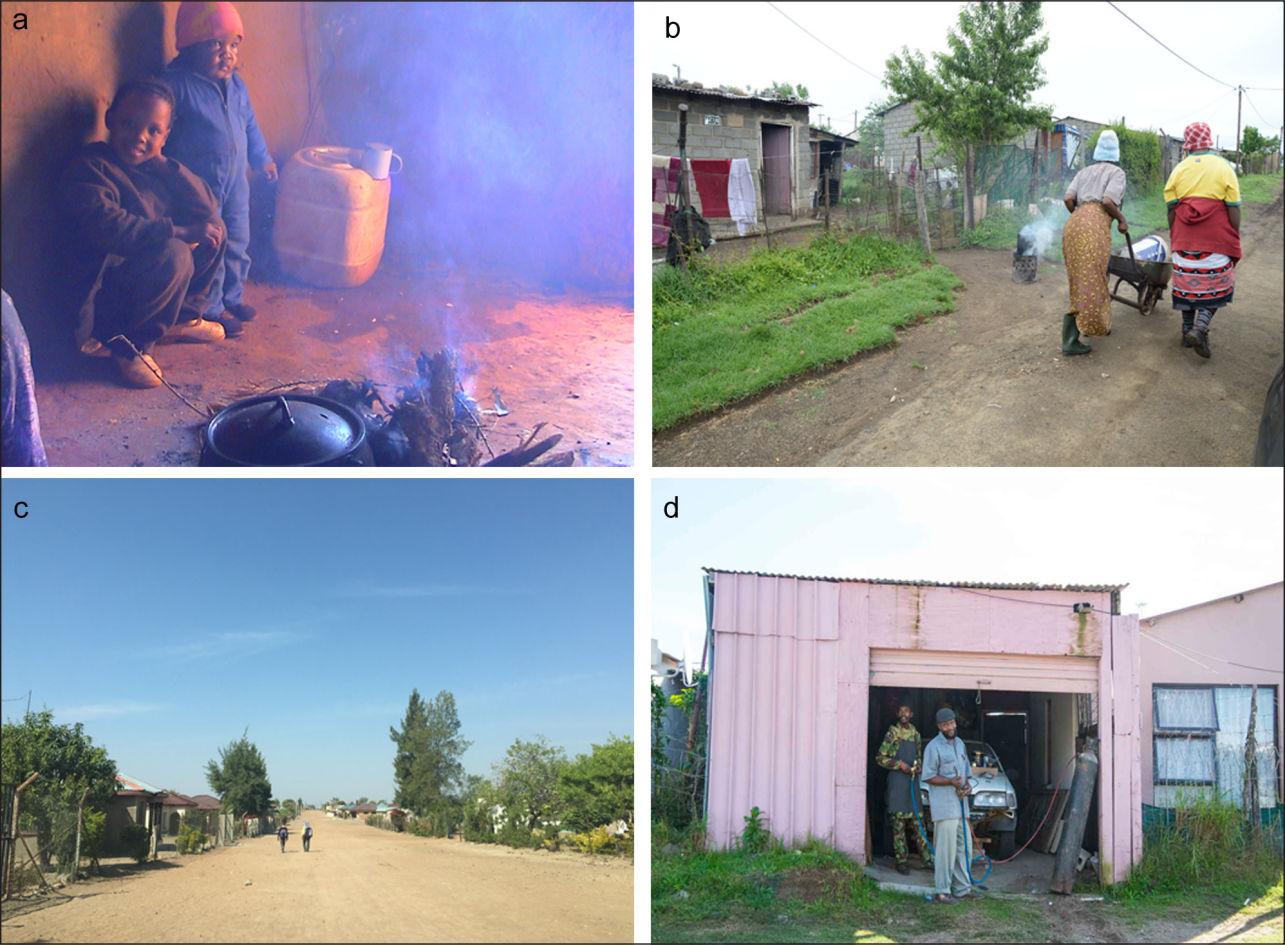
**a)** Household air pollution, **b)** domestic burning, **c)** unpaved roads and **d)** cottage industrial activities are all sources of air pollution [Photograph credits, (a) Brendon Barnes, (b) Angela Mathee, (c) Caradee Y Wright and (d) Angela Mathee].

Sources of human exposure to air pollution in Africa include anthropogenic and natural sources and occur in urban, rural, industrial and residential settings. The main contributors are industry, power generation, agricultural burning, transport and traffic, the combustion of wood, coal, paraffin and dung for household energy needs (Figure 1a, b), unpaved roads and burning of household solid waste in areas not provided with regular residential waste collection services. Desert dust and wildfires are sources of particulate matter of natural origin [2].

Certain vulnerable groups may be simultaneously exposed to air pollution from multiple sources – sometimes at highly elevated concentrations [3]. For example, people living in informal settlements without a connection to the electricity grid, located close to mine tailings facilities or industrial sites, and in areas with unpaved roads (Figure 1c). In countries with high unemployment, many households generate livelihoods in the informal sector, including cottage industries (Figure 1d) such as vehicle spray painting, which may lead to increased levels of air pollutants in the immediate vicinity that frequently exceed the air quality guidelines of the World Health Organization [4,5]. A study undertaken in Accra, Ghana showed that biomass burning accounted for 39–62% of total PM_2.5_ mass in the cooking area, road dust and vehicle emissions comprised 12–33% of PM_2.5_ mass, and solid waste burning was also a significant contributor to household PM_2.5_ in low-income settlements [6]. Patriarchal systems and household power dynamics may play a role in women and children being particularly vulnerable to exposure to noxious air pollutants [7].

### The Challenge of Addressing Air Pollution in Africa

Tackling air pollution in Africa is undoubtedly important but constitutes a uniquely complex challenge. Unlike many OECD countries, African efforts to curb exposure to air pollution need to be implemented alongside actions to address competing health and economic challenges, including poverty and inequality, a process of rapid urbanization currently underway, housing shortages, unsafe water, inadequate sanitation and major epidemics such as HIV/AIDS. Poor people are exposed to higher concentrations of air pollutants, have access to inferior health services, and tend to suffer disproportionately from the effects of air pollution [5]. In settings of poverty, where safe energy alternatives are not available, legislation to curb household solid fuel combustion would place additional hardship and financial burdens on poor households. While being home to 16% of the world’s population, only 3% of the world’s vehicle fleet is found in Africa [1,8]. Traffic-related air pollution may gain in importance as a source of air pollution, given the predicted population and income increases in Africa in the coming decades, and the process of rapid urbanization currently underway. The current African population of approximately 1.2 billion is predicted to rise to 2.5 billion by 2050, and to 4.4 billion (or 40% of the world’s population) by 2100 [8].

In this regard, a fundamental concern is that air quality monitoring capacity in Africa is weak. One study reported that only 41 cities across 10 African countries measured ambient air pollution levels, and knowledge of the sources and pathways of human exposure to air pollution across is limited to well-resourced countries, providing a weak base for policy development and priority setting [9].

### Harnessing opportunities from the Global Statement on Air Pollution

As stated in the July 2019 Air Pollution and Health Statement jointly issued by the Academy of Sciences of South Africa (ASSAf), the Brazilian Academy of Sciences (ABC) and the German National Academy of Sciences Leopoldina as well as both the US National Academies of Medicine and Science (USNAM and USNAS), “the costs of air pollution to society and the economies of low- and middle-income countries are enormous” and “can undercut sustainable development” [10]. African countries thus have much to lose from limited action on air pollution, but much more to gain from heeding the Joint Call for investment in air pollution reduction.

### Signs of Hope and Success

**Box 1: Case study**

The Integrated High-Speed Train Network is a flagship project of the African Union’s Agenda 2063. The project aims to connect all African capitals and commercial centers through an African High-Speed Train Network thereby facilitating the movement of goods, factor services and people. The increased rail connectivity holds the potential for reducing transport costs, relieving traffic congestion and lowering traffic emissions [11]. Emerging low-cost air quality monitoring technologies also provide hope for more extensive air quality monitoring systems and the generation of improved information for future decision-making.

While the challenges are complex, some novel solutions are emerging to overcome air pollution in some African countries. In South Africa, at a regional scale, the Greenhouse Gas and Air Pollution Interactions and Synergies or ‘GAINS’ model is a useful framework being considered to identify strong linkages between air quality and climate-relevant measures [12]. The results would provide evidence for improving understanding of the cost-efficiency of air pollution policies in line with the Statement’s recommendation to ‘*identify co-benefits among policy instruments*’. Renewable energy technologies that are increasingly deployed at grid- and household-level present another opportunity for policy co-benefits by reducing air pollution and greenhouse gas emissions from large fossil fuel-fired power stations and household fuel burning.
